# Hepatic hemangioma -review-

**Published:** 2015

**Authors:** N Bajenaru, V Balaban, F Săvulescu, I Campeanu, T Patrascu

**Affiliations:** * Department of Surgery II, “Dr. Carol Davila” Central Military Emergency University Hospital, Bucharest, Romania; ** Gastroenterology Clinic, “Dr. Carol Davila” Central Military Emergency University Hospital, Bucharest, Romania; *** Department of Surgery, “Sf. Maria” Clinical Hospital, Bucharest, Romania; **** “I. Juvara” Department of Surgery, “Dr. I. Cantacuzino” Clinical Hospital, Bucharest, Romania

**Keywords:** hepatic hemangioma (HH), conventional ultrasonography (US), contrast-enhanced ultrasonography (CEUS), ultrasonography contrast agent (UCA), computer tomography (CT)

## Abstract

Hepatic hemangiomas are benign tumors of the liver consisting of clusters of blood-filled cavities, lined by endothelial cells, fed by the hepatic artery. The vast majority of HH are asymptomatic, most often being discovered incidentally during imaging investigations for various unrelated pathologies. Typical hemangiomas, the so-called capillary hemangiomas, range from a few mm to 3 cm, do not increase in size over time and therefore are unlikely to generate future symptomatology. Small (mm-3 cm) and medium (3 cm-10 cm) hemangiomas are well-defined lesions, requiring no active treatment beside regular follow-ups. However, the so-called giant liver hemangiomas, of up to 10 cm (most commonly) and even 20+ cm in size (according to occasional reports) can, and usually will develop symptoms and complications that require prompt surgical intervention or other kind of therapy.

HH belong to the class of hepatic “incidentalomas”, so-called because they are diagnosed incidentally, on imaging studies performed as routine examinations or for other reasons than the evaluation of a possible liver mass. Less than half of HH present with overt clinical symptoms, consisting, most often, of upper abdominal pain (this is usually the case for large lesions, which cause the distension of Glisson’s capsule).

Hepatic hemangiomas require a careful diagnosis to differentiate from other focal hepatic lesions, co-occurring diagnoses are also possible.

## Introduction

Hepatic hemangiomas are benign tumors of the liver consisting of clusters of blood-filled cavities, lined by endothelial cells, fed by the hepatic artery. The vast majority of HH are asymptomatic, most often being discovered incidentally during imaging investigations for various unrelated pathologies. Typical hemangiomas, the so-called capillary hemangiomas, range from a few mm to 3 cm, do not increase in size over time and therefore are unlikely to generate future symptomatology. Small (mm-3 cm) and medium (3 cm-10 cm) hemangiomas are well-defined lesions, requiring no active treatment beside regular follow-ups. However, the so-called giant liver hemangiomas, of up to 10 (most commonly) and even 20+ cm in size (according to occasional reports) can, and usually will develop symptoms and complications that require prompt surgical intervention or other kind of therapy [**1**].

We present a survey of the recent literature on hepatic hemangiomas with particular attention to new developments in diagnostic imaging investigations and liver surgical techniques. The subject has growing importance, as methods of diagnosis have been refined and can better identify their incidence and prevalence in the general population - hemangiomas that would have gone undetected in the past, are now being diagnosed, while modern techniques in liver surgery could make possible the treatment of cases that were previously thought of as surgically unapproachable.

**Terminology:** hepatic hemangioma/ liver hemangioma/ cavernous hemangioma

Hepatic hemangioma (HH) is the most common benign liver tumor. It consists of blood-filled cavities fed by the hepatic arterial circulation, with walls lined by a single layer of endothelial cells, a veritable chaotic entanglement of distorted blood vessels confined to a region as small as a few mm and as large as 10 cm, 20 cm [**3**,**4**,**12**] and even 40 cm [**9**]. The frequency is higher among adults, with a prevalent age at the initial diagnostic in the range of 30-50 years [**2**]. Literature places the HH incidence at 0.4% to 20% of the total population [**3**,**5**]. At necropsy, the frequency is of 0.4 to 7.3% [**3**,**6**], all the authors agreeing with an incidence of over 7%. The HH prevalence in the general population varies greatly, most often being discovered incidentally during imaging investigations for various unrelated pathologies. Regarding sex distribution, it seems that women are more susceptible, as confirmed by all pertaining studies, with a reported 4.5:1 to 5:1 ratio of female to male cases [**7**,**8**]. Most often, HH are mono-lesions but multiple-lesions are possible; they account for 2.3% [**2**] and up to 20-30% [**4**] of the cases, depending on the source. At the initial diagnosis, the majority of HH measure below 3 cm in size, the so-called capillary hemangiomas; of these, only 10% undergo a size increase with time, for reasons still unknown. The next size class covers lesions between 3 cm and 10 cm in size, referred to as medium hemangiomas. Lastly, giant or cavernous hemangiomas measure up to 10 cm, with occasional literature reports of giant HH reaching 20-40+ cm in size [**9**]. Location-wise they are most often found in the right liver lobe, often in segment IV, often marginal [**10**].

## Etiopathogeny

The cause of HH is not known, it may be congenitally determined, there are researchers who reported cases of HH running in families, suggesting a possible genetic connection, others with mesenchymal origin, still, others considered congenital hematoma in some articles. Infantile HH can be diagnosed prenatally or in childhood. Reported frequency at 1 year is of 5-10%, normally they regress in size over time, but not always.

## Symptoms

In most situations, HH do not show any signs and/or symptoms, most likely being discovered incidentally during imaging investigations for other unrelated conditions. If symptoms do occur, they are nonspecific, common to many other diseases, especially of digestive origin. Pain in the right upper hemiabdomen is the most common complaint; others include decreased appetite, premature satiation sensation, nausea, vomiting, abdominal discomfort: sense of fullness, postprandial bloating, early or late. These symptoms can indicate the presence of a hemangioma or can be caused by other disorders independent of the presence of HH [**5**]. Physical exam can detect hepatomegaly and very rarely a palpable mass. HH show **complications** depending on size and location: **inflammatory**, acute (fever) and chronic; **mechanical:** *rupture*, spontaneous or traumatic: intra-abdominal mass disruption trauma, or marginal trauma when located in the proximity of the costal margin, hence more exposed to trauma, *compression* of adjacent structures: stomach, resulting in gastric obstruction (early feeling of fullness), bile ducts, leading to jaundice, haemobilia, *volvulus / torsion / infarction* for pedunculated HH; **bleeding:** intratumoral or intraperitoneal, with or without consumptive coagulopathy: Kassalbach-Merritt syndrome (HH giant, thrombocytopenia, intravascular coagulation), Osler-Rendu-Weber disease (hereditary telangiectasia: multiple smaller HA on face, tongue, jugal mucosa, gastrointestinal tract, liver), Klippel-Trenaunay syndrome (congenital hemiatrophy nevus flammeus, hemi-mega-encephalopathy), Von Hippel-Lindau disease (cerebral, retinal, pancreatic hemangioma); **degenerative:** thrombosis, hyalinization, progressive fibrosis and sclerosis becoming central scar. Particular cases of HH: pedunculated, calcified, on liver steatosis, on cirrhotic liver, with massive arteriovenous shunt, complicated with heart failure. Co-pathologies associated with hepatic hemangioma include: most frequently hemangiomatosis, focal nodular hyperplasia, and angiosarcoma.

Predisposing factors of complications of HH: adulthood, chronic medication use (such as steroid use, can accelerate the development of an existing HH), female sex: estrogen therapy, use of oral contraceptives (increase the risk or increase the size, discontinuing contraceptive regimen can lead to lesion regression, but not necessarily); pregnancy and multiparity (by disrupting estrogen and progesterone hormone levels, leading to an increase in size of a preexisting HH); replacement therapy for menopausal symptoms; ovarian stimulation treatment with clomiphene citrate and human chorionic gonadotropin. Genetic gene penetrance or sex hormone proliferative factors could also be an explanation.

Physical exam does not come with notable modifications, as do not routine laboratory tests, including liver chemistry [**6**]. Hypofibrinogenemia occurs due to intratumoral fibrinolysis, while thrombocytopenia is associated with large lesions, being a consequence of spleen sequestration and destruction. Tumor markers: alpha-fetoprotein (AFP), CA 19-9 (carcinogenic antigen 19-9) and carcinogenic embryonic antigen (CEA) within normal limits advocate for the benign nature of the lesion.

## Diagnosis

HH is usually diagnosed incidentally on imaging studies performed as routine examinations or for other reasons than the evaluation of a possible liver mass. Less than half of HH present with overt clinical symptoms, consisting of upper abdominal pain, sensation of weight or fullness (this is usually the case for large lesions, which cause the distension of Glisson’s capsule) [**11**].

Imaging diagnosis of HH includes conventional ultrasound (US, B-mode and Doppler), contrast-enhanced ultrasound (CEUS), contrast-enhanced computed tomography (CT), magnetic resonance imaging (MRI), angiography and nuclear scans (scintigraphic studies with Technetium-99m labeled red blood cells), offering good specificity for the diagnosis of HH. These are used in order to differentiate HH from other vascular tumors, benign lesions (adenoma) or malignant ones (HCC, metastasis, dysplastic nodules).

**Ultrasound (US)**

Due to its wide availability, lack of irradiation and reproducibility, ultrasound is usually the first diagnostic step for HH. The main limitation of US is that it is highly operator and patient-dependent. On conventional ultrasound, HH appears as a hyperechoic homogenous nodule, with well-defined margins and posterior acoustic enhancement [**12**]. Moreover, on follow-up exams or while comparing the current scan with the previous ones, HH usually does not change in size [**13**]. The hyperechoic pattern on US is explained by the histology of HH – the hyperechogenicity is a result of the numerous interfaces between the endothelial lined sinuses composing the HH and the blood within them. This hyperechoic appearance is usually the case for small HH; larger lesions, because of possible necrosis, hemorrhage or fibrosis can appear inhomogeneous, with mixed echogenicity (hypo- and hyperechoic). Lesions that have such echo patterns are labeled as atypical HH. On Doppler US, most HH show minimal or no Doppler signal [**14**].

 However, not every hyperechoic mass should be labeled as HH. This echo pattern can also be seen with other benign (adenomas) or malignant pathology (hepatocellular carcinoma, metastasis). As discussed, stable findings on serial examinations are a very reliable sign in clinical practice for benign disease. US has a good accuracy in differentiating HH from malignant hyperechoic masses (sensitivity of 94.1% and specificity of 80.0% for lesions under 3 cm diameter). The absence of lesion blood flow in HH on Doppler US is also a reliable sign for the differential diagnosis with hepatocellular carcinoma (HCC), which frequently has intra- or peritumoral vascularity [**15**]. In hypoechoic lesions, a peripheral echogenic rim can suggest HH. In contrast, a peripheral perilesional hypoechoic rim, known as the “target sign”, is rarely seen in HH [**14**]. Another differential diagnosis to be considered is focal nodular hyperplasia (FNH), which has the characteristic “spoke-wheel sign” [**16**]. Caution should be kept in mind when assessing the fatty liver, in which a typical hemangioma can seem hypoechoic relative to the intense hyperechoic liver parenchyma. 

**Contrast-enhanced ultrasound (CEUS)**

CEUS is a good tool for a more specific diagnosis of HH than conventional US. Using microbubbles that better delineate the microvasculature, CEUS generates real-time perfusion imaging within the lesion similar to the vascularity pattern seen in CT scans. This is especially useful for the differential diagnosis of a liver nodule, being able to accurately discriminate a HH from adenomas, FNH, HCC or metastasis. The typical HH shows peripheral nodular enhancement in the arterial phase with complete (but sometimes incomplete) centripetal filling in the portal venous and late phases [**17**]. This characteristic enhancement pattern has a sensitivity of 98% for histologically proven HH [**17**]. Besides this typical appearance, one should be aware that a HH can rarely have a centrifugal enhancement [**18**,**19**].

Two second generation ultrasonography contrast agents (UCA) have been approved for ‎use in Romania: SonoVue® (sulfur hexafluoride) for hepatic applications, introduced in 2001 by Bracco SpA, Milan, Italy and licensed for liver imaging in Europe, China, India, Korea, Hong Kong, New Zealand, Singapore and Brazil, and Optison® - designed as a contrast agent in echocardiography. Two other UCA are in common use today: Definity/ Luminity® (octafluoropropane–perfluten) available since 2001 in Canada and Australia, and Sonazoid® (perfluorobutane) [**21**], introduced in 2007 in Japan and now in South Korea and Norway [**22**].

Usually a phospholipid shell stabilizes gas bubbles. The microbubbles ‎used in CEUS have a number of characteristics that significantly enhance ultrasound signal intensity: they are sufficiently small to escape the pulmonary capillary barrier (typically a few micrometers), but at the same time too large to cross the vascular endothelium, therefore they remain intravascular for the duration of the exam [**22**].

**Fig. 1a:**
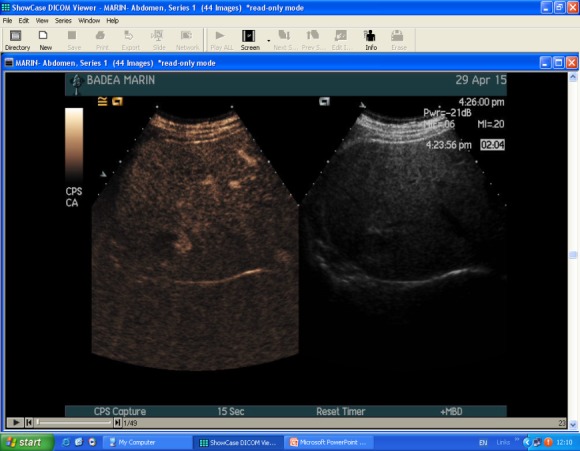
CEUS split screen image: left - native US, right - with SonoVue® UCA

**Fig. 1b:**
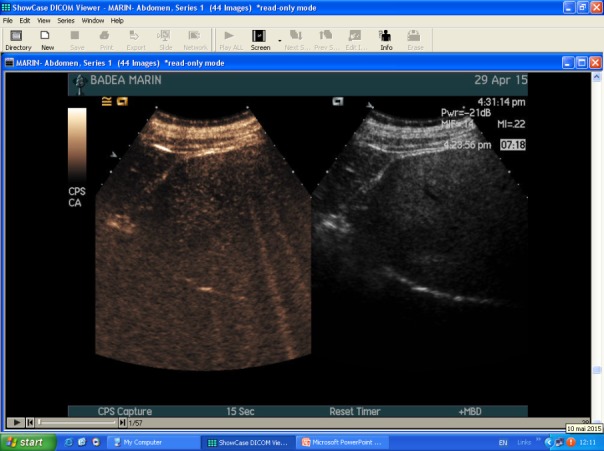
CEUS split screen image: left - native US, right - with SonoVue® UCA

The UCA comes as a powder and a solvent, upon mixing it becomes a milky liquid and is administered as a bolus intravenous injection immediately followed by a flush bolus injection of 10 ml saline solution [**22**].

CEUS has many advantages, the major ones include the real-time examination and delivery of results, ability to follow multiple lesions simultaneously, repeatability, re-injection and absence of contraindications (iodine allergy, hepatic failure, renal failure) [**23**,**24**]. The accuracy of CEUS is reduced in patients with fatty liver or deep situated lesions. Since UCA is non-ionizing and non-toxic [**20**], CEUS makes feasible simultaneous investigation of multiple lesions, which require reinjection of contrast material **Fig. 1a**-**b**. 

The typical HH appears on CT scans as a hypodense, well-defined lesion, which after contrast injection shows peripheral nodular enhancement with progressive centripetal homogeneous filling. This particular pattern cannot be highlighted in very small lesions of less than 5 mm, which can be difficult to characterize. As with CEUS, atypical HH can show different enhancement patterns on CT [**23**,**25**,**26**]. Non-enhancing intralesional spots can occur with fibrosis, thrombosis or necrosis, leading to a heterogeneous presentation. HH that are homogenous and rapidly enhancing in the arterial phase can be mistaken for hypervascular tumors. In patients with severe fatty infiltration of the liver, HH can appear hyperdense relative to the adjacent liver parenchyma. The main limitations of the CT are radiation and the use of iodine contrast media (which can cause contrast-induced nephropathy) **Fig. 2a**-**c**.

**Fig. 2a:**
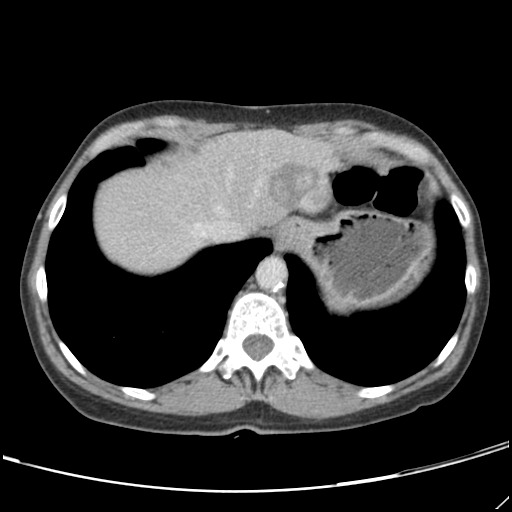
CT – axial section

**Fig. 2b:**
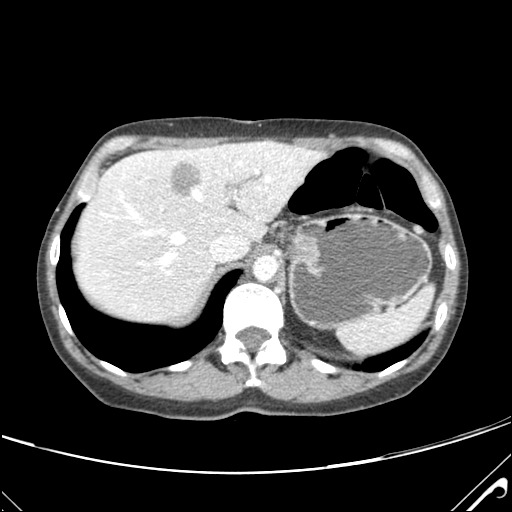
CT – axial section

**Fig. 2c:**
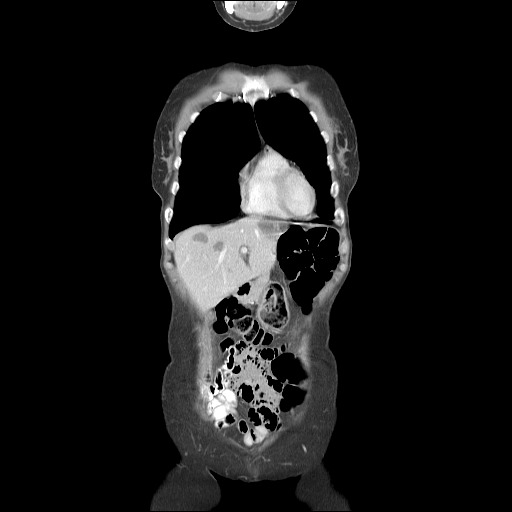
CT – coronal section

**Fig. 3:**
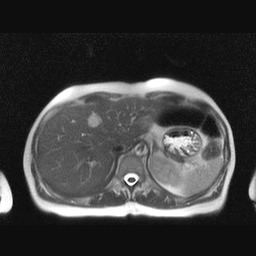
MRI

On **MRI**, the typical appearance is a well-demarcated, homogenous lesion, hypointense on T1-weighted images and hyperintense on T2-weighted images, the “cotton-wool” aspect [**27**]. Because both, malignancy and HH are hyperintense on T2-weighted images, the differentiation is done by increasing the echo time (TE): while the signal from malignant lesions tends to decrease, the one from HH increases [**28**]. Diffusion-weighted images are also useful in differentiating HH from malignant lesions. UCA is gadolinium-based in MRI and can be used in patients with allergy to iodinated contrast agents or renal failure, for whom CT is contraindicated [**26**] **Fig. 3**.

**Fig. 4:**
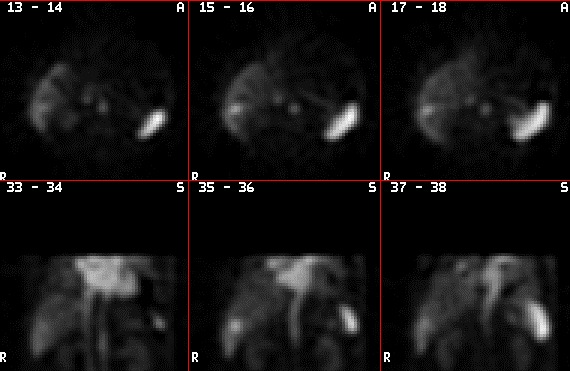
Scintigraphy

**Technetiu-99m labeled Red Blood Cell scintigraphy**

Tc-99m RBC scintigraphy is a noninvasive method, which provides the most specific diagnosis of hepatic hemangioma. The characteristic, diagnostic presentation of HH on Tc-99 labeled RBC images is perfusion/ blood pool mismatch: decreased perfusion on early dynamic images and a gradual increase in activity on blood pool images over time. The lesion appears “cold” in the early dynamic phase and finally intense in the late phase, 1-2 hours following Tc-99m injection. Sensitivity is strongly size-dependent, especially at the small end of the range: 17-20 % for the detection of lesions less than 1 cm in size, 65-80% for lesions between 1 cm and 2 cm, and virtually 100% for those larger than 2 cm. The specificity of Tc-99m labeled RBC scintigraphy with SPECT (Single Photon Emission Computed Tomography) remains at 100% over the entire size range. Although it has very high sensitivity and specificity, scintigraphy is always followed by either a CT or a US exam to establish location, shape and multiplicity of the lesion. Reduced availability, high cost and length of procedure, its irradiating nature and a variety of feasible competing imaging technologies led to its abandonment as a diagnostic method for HH, [**10**] **Fig. 4**.

**Table 1:** Accuracy of different diagnostic methods for HH

*Imaging method*	*Sensitivity*	*Specificity*
Ultrasound	96,9%	60,3%
CEUS	98 %	100%
CT	98,3%	55%
MRI	100%	85,7%
Tc-99m scintigraphy	75%	100%
Adapted after [**34**].		

**Angiography**

Selective or ultra-selective hepatic angiography has the highest specificity for the pattern of HH, but it is not used for diagnosis of such lesions because of the availability of the noninvasive methods previously described [**29**].

**Histology sampling**

Due to its vascular nature, biopsy with histological sampling has a great risk of hemorrhage (especially in large, subcapsular lesions), including mortality [**30**,**31**]. Besides this risk, the diagnostic yield is not as high as expected: in a study with 36 patients, the diagnostic material was obtained in only 21 of them [**32**]. Biopsy is thus reserved for extremely atypical lesions, with equivocal features on imaging [**33**].

**Histological examination**

On hematoxylin-eosin staining microscopy, HH appear as dilated vascular channels lined by a single layer of endothelial cells. Complications of HH include necrosis, thrombin, sclerosis or calcification. No malignant transformation has been reported [**33**].

## Treatment

Most of HH are small and asymptomatic at the time of diagnosis and the evolution is relatively stationary. There is no data in literature to advocate for malignant transformation. A management by supervision through imaging methods at every 6 months or annually in order to assess the scale of the development over time is considered sufficient. Long-time observation is necessary in patients who have new-onset pain or are unresponsive to analgesics, who are getting estrogen therapy, during pregnancy and mandatory for large HH. According to existing data, there is no known pharmacological therapy able to reduce the size of HH. Anti-angiogenic therapy with bevacizumab (a monoclonal antibody capable of inhibiting endothelial growth factor activity) was considered, without confirmation.

Indications for surgery are rapid growth in size, pain despite analgesics or both. Nowadays, the following are reconsidered as absolute indications for surgery: dimensions, localization or risk of intratumoral thrombosis, rupture or other complications.

Apart from surgical modalities, there are other options available to treat symptomatic HH, such as arterial embolization or radiofrequency ablation. 

Surgical management includes segmental resections, lobectomy or enucleation of the hemangioma, by open surgery or laparoscopy [**6**].

**Fig. 5:**
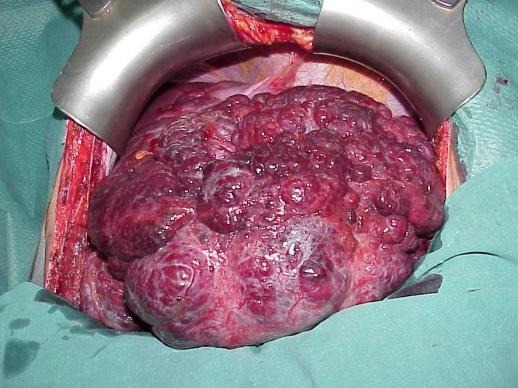
Enucleation of giant HH **Adapted after** Campeanu I. – personal collection

**Surgical resection:** segmental resection or enucleation? **Fig. 5**.,**6**. For elective surgery, the choice is dictated by size and location, but also by the preference and technical skill of the surgeon. More and more, surgeries are done in a minimally invasive manner - laparoscopically and, recently, with robotic surgery supporting the technical skills. For all techniques, the postoperative morbidity is minimal, HH rarely recur after surgery [**35**]. Right or left hepatectomy is indicated for large masses that occupy the entire lobe [**36**-**38**]. Literature reports mortality for emergency procedures. 

**Fig. 6:**
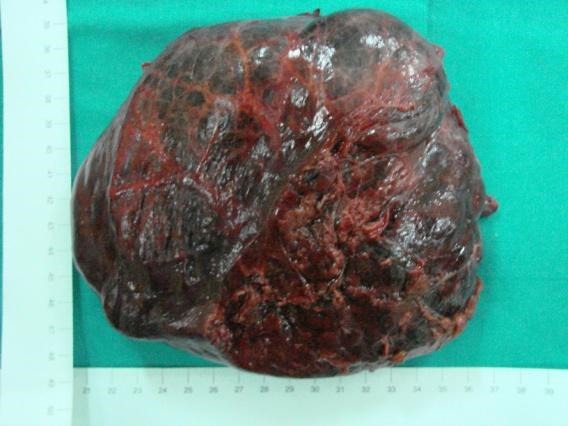
Excised HH specimen **Adapted after** Campeanu I. – personal collection

Selective or supra-selective **angiographic embolization** with polyvinyl alcohol or other substances by catheterization of the hepatic arteries can lead to tumor reduction. The procedure is effective in restricting and reversing tumor growth only for hemangiomas that have a clearly identified arterial blood supply [**9**]. The long-term success rate of arterial embolization is not well-studied [**39**]. **Portal vein embolization** (PVE) is now routinely used prior to resection to increase the residual (post-surgery) viable liver parenchyma, providing more favorable conditions for the elective major surgery, and minimizing the risk of complications [**40**].

Selective **surgical transhepatic ligation** of the HH major feeding vessels can successfully reduce intratumoral shunt which would have led to congestive heart failure [**41**,**42**].

**Radiofrequency ablation**, executed laparoscopically or percutaneously under ultrasound control has been used for pain control in small studies [**43**].

**Irradiation of the liver**. Liver irradiation with a dose of 15-30 Gray in 15-22 sessions for a few weeks results in tumor regression and has minimal secondary morbidity [**44**].

**Orthotopic liver transplantation** is indicated for large or diffuse bilateral lesions. Only a few cases have been reported in literature.

## Discussion and Conclusion

While most people with HH show no sign or symptom, and most HH are non-progressing and do not require treatment, there is a small number of cases with rapid volumetric growth or complications, which prompt for appropriate therapy. The results of clinical and laboratory investigations to date, mostly for imaging techniques, have demonstrated that for small HH, regular follow-up is enough. For cavernous HH, the evolution is unpredictable and often unfavorable, with serious complications requiring particular surgical expertise in difficult cases. Hepatic hemangiomas require a careful diagnosis to differentiate from other focal hepatic lesions, co-occurring diagnoses are also possible.
